# Integrating cryoablation with immunotherapy in non-small cell lung cancer: mechanisms, clinical evidence, and translational barriers

**DOI:** 10.3389/fonc.2026.1869239

**Published:** 2026-07-31

**Authors:** Chulan Yang, Xinan Long, Yangyang Ma, Lizhi Niu

**Affiliations:** 1 Central Laboratory, Guangzhou Fuda Cancer Hospital, Guangzhou, China; 2 Department of Oncology, Guangzhou Fuda Cancer Hospital, Guangzhou, China

**Keywords:** combination therapy, cryoablation, immunotherapy, NSCLC, tumor microenvironment

## Abstract

Despite major advances in targeted therapy and immune checkpoint blockade, advanced NSCLC still has poor long-term outcomes. Only a minority of patients achieve durable benefit, and many eventually experiencing disease progression. Local ablation techniques, particularly cryoablation, directly destroy tumors and remodel the local immune microenvironment by inducing immunogenic cell death and releasing tumor antigens and damage-associated molecular patterns (DAMPs). These processes may contribute to systemic antitumor immunity and are associated with a more inflamed tumor immune microenvironment. This review summarizes the biological mechanisms underlying the potential synergy between cryoablation and immunotherapy, particularly immune checkpoint inhibitors. Cryoablation has been proposed to act as an *in situ* vaccination strategy by preserving native tumor antigens and enhancing antigen presentation and T-cell priming. These effects may in turn trigger systemic antitumor immune responses beyond the treated site. The article summarizes preclinical and clinical evidence supporting this combined approach. Available studies suggest that cryoablation combined with immunotherapy may improve tumor control and survival outcomes in selected NSCLC settings. However, current clinical evidence remains heterogeneous and is still limited in scale. Finally, we discuss key challenges, including treatment sequencing, biomarker development, technical standardization, and long-term safety, and outline future directions for prospective and biomarker-informed clinical studies. This review highlights the potential role of cryoablation as an adjunct to immunotherapy in selected NSCLC settings, while emphasizing the need for further prospective validation.

## 1 Introduction

NSCLC remains the leading cause of cancer-related mortality worldwide, representing over 85% of all lung cancer cases. Despite significant advances in systemic therapy, the prognosis for patients with advanced (Stage IV) disease has historically been poor, with traditional therapeutic modalities including surgical resection, chemo-radiotherapy, and molecularly targeted therapies frequently constrained by restricted eligibility, systemic toxicity, or the inevitable emergence of therapeutic resistance (1–4).

The advent of immune checkpoint inhibitors (ICIs), particularly those targeting the PD-1/PD-L1 axis, has substantially changed the management of advanced NSCLC (5). Landmark phase 3 trials with 5-year follow-up have demonstrated substantially improved long-term survival: for patients with PD-L1 tumor proportion score (TPS) ≥50%, first-line pembrolizumab monotherapy achieved a 5-year overall survival (OS) rate of 31.9% versus 16.3% with chemotherapy (6); In patients with non-squamous NSCLC irrespective of PD-L1 expression, pembrolizumab plus chemotherapy yielded a 5-year OS rate of 19.4% versus 11.3% with chemotherapy alone (7). However, the clinical utility of ICI monotherapy is hindered by a relatively low objective response rate (ORR) of approximately 20% to 30% (8). This limited efficacy is largely attributed to the “cold” tumor microenvironment (TME), characterized by sparse T-cell infiltration, an accumulation of immunosuppressive cells (e.g., regulatory T cells), and dense biochemical barriers that facilitate immune evasion (9). Consequently, there is a strong rationale for identifying synergistic strategies capable of converting immunologically “cold” tumors into more inflamed and treatment-responsive lesions.

Local ablation techniques have attracted increasing attention as potential modulators of the tumor immune microenvironment. Unlike radiofrequency or microwave ablation, which rely on heat-based tissue destruction and may alter antigenic structures, cryoablation uses freeze–thaw cycles that may better preserve tumor-associated antigens and support immune recognition (10). This mechanism may contribute to an *in situ* vaccination-like effect, a process in which local tumor destruction releases tumor antigens and immunostimulatory signals that stimulate systemic antitumor immunity. Specifically, cryoablation has been reported to induce features consistent with immunogenic cell death (ICD) and the release of damage-associated molecular patterns (DAMPs), which may promote antigen-presenting cell activation and tumor-specific T-cell priming, with the potential to contribute to systemic antitumor responses in distant lesions (11). In parallel with these preclinical and mechanistic insights, a growing number of clinical studies have begun to evaluate the safety and efficacy of combining cryoablation with immunotherapy in NSCLC, although the available evidence remains predominantly retrospective and heterogeneous (12).

Compared with some thermal ablation modalities, cryoablation may better preserve antigenic structures and may generate a distinct immunogenic context, which could favor synergy with immunotherapy (13, 14). Given its dual role in local tumor destruction and potential immune modulation, the combination of cryoablation and immunotherapy has emerged as a promising area of translational interest (15, 16). In this review, we summarize the biological basis of cryoablation-induced immune modulation, evaluate the current preclinical and clinical evidence for combining cryoablation with immunotherapy in NSCLC, and discuss the major barriers to clinical translation.

## 2 Mechanisms and immunological effects of cryoablation

### 2.1 Overview of cryoablation technology

Cryoablation, also referred to as cryotherapy or cryosurgery, is a minimally invasive image-guided technique that uses extremely low temperatures to destroy target tissue *in situ*. Its modern clinical application began in the 1960s. Advances in imaging guidance technologies, including ultrasound, CT, and MRI, together with improvements in argon–helium cryosystems, have expanded the application of cryoablation across a range of solid tumors, including pulmonary malignancies (17–22).

The fundamental principle underlying cryoablation is predicated on the Joule-Thomson effect (23). During treatment, high-pressure argon gas (for cooling) and helium gas (for rewarming) are cyclically delivered to the target tissue via a hollow probe inserted through percutaneous puncture (22). Argon undergoes rapid expansion at the probe tip, absorbing heat and forming low-temperature ice crystals (24). Subsequently, the switch to helium results in active rewarming of the tissue. The freeze–thaw process is central to cryoablation, and while early protocols generally employed two cycles, contemporary evidence indicates that triple freeze–thaw cycles are associated with superior local tumor control and have become the standard of care in many high-volume institutions (14, 25–27).

### 2.2 Mechanisms of tumor destruction and immune activation

#### 2.2.1 Direct cytotoxic effects on tumor cells

Direct cytotoxic effects result from multiple physicochemical injuries during freeze-thaw cycles. Intracellular and extracellular ice crystal formation directly disrupts cell membranes and causes mechanical damage. Extracellular ice also leads to cellular dehydration, shrinkage, and abnormally high intracellular solute concentrations, a phenomenon often referred to as “solute toxicity”. Repeated freeze-thaw cycles synergistically enhance tumor cell killing compared to single-cycle therapy (28, 29).

#### 2.2.2 Indirect damage to blood vessels

Beyond direct cellular destruction, cryoablation damages the tumor microvasculature. Low temperatures injure vascular endothelium, initiating platelet aggregation and microthrombi formation, which lead to microcirculatory embolism. Subsequent prolonged ischemia and hypoxia induce secondary coagulative necrosis within the tumor (30, 31).

#### 2.2.3 Cryoablation-induced immune activation and *In situ* vaccination

**Figure 1:**
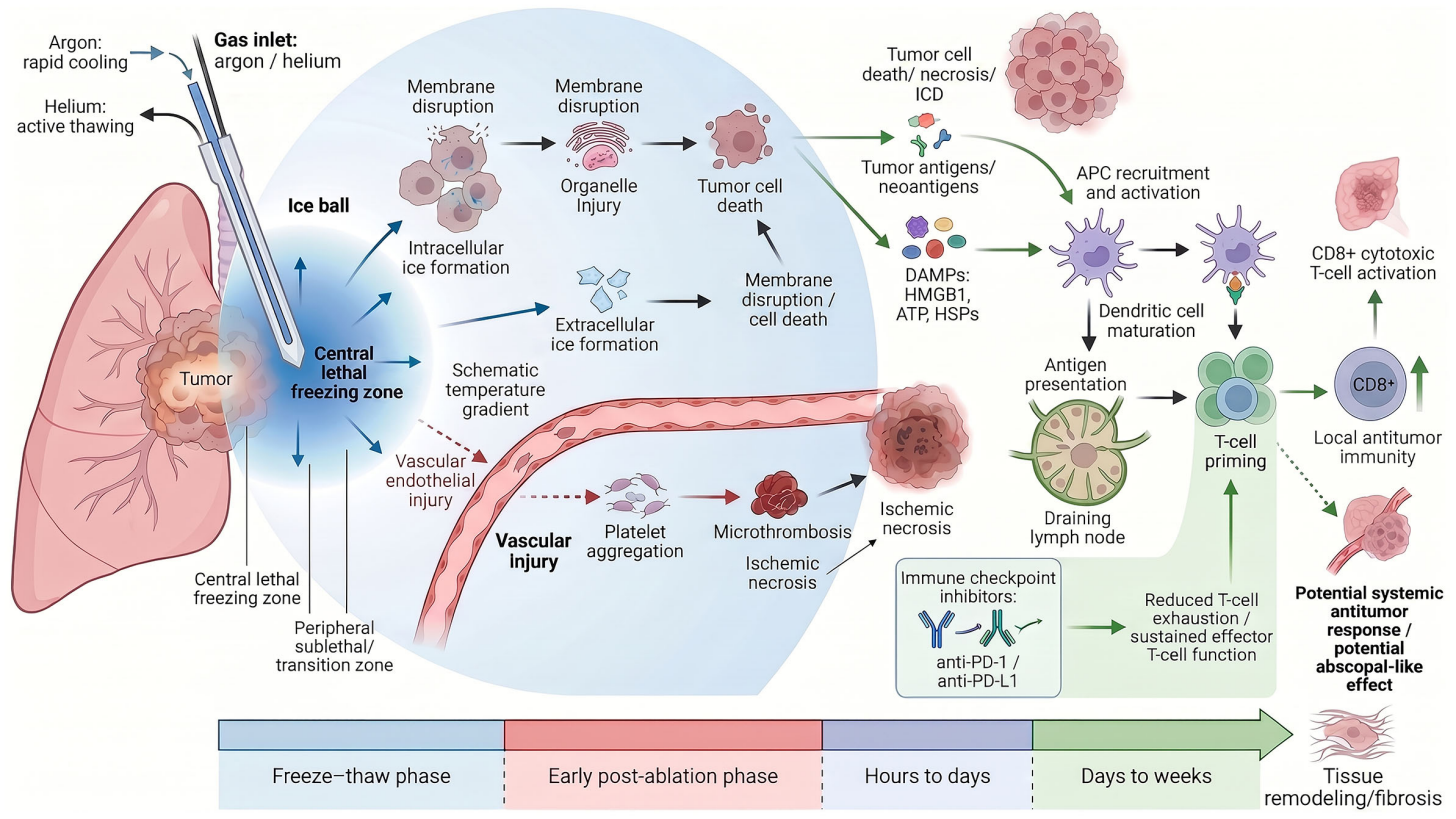
Mechanisms of cryoablation-induced tumor destruction and immune activation in lung cancer. The cryoprobe is connected to a gas inlet that delivers argon for rapid cooling and helium for active thawing. During the freeze–thaw phase, intracellular and extracellular ice formation directly injures tumor cells by disrupting cellular membranes and generating osmotic stress, leading to cellular dehydration, membrane damage, and tumor cell death. The ice ball contains a central lethal freezing zone and a peripheral sublethal or transition zone, reflecting a schematic temperature gradient rather than fixed procedural thresholds. During the early post-ablation phase, vascular endothelial injury, microthrombosis, and ischemic necrosis further contribute to tumor destruction. Over subsequent hours to days, tumor antigens and damage-associated molecular patterns are released, promoting antigen-presenting cell activation, T-cell priming, and potential systemic antitumor immune responses. Tissue remodeling and fibrosis may occur during the later post-ablation phase.

In contrast to thermal ablation, which may denature tumor antigens and reduce their immunogenicity, cryoablation may better preserve antigenic structures and thereby support downstream adaptive immune responses. In addition to direct tumor destruction, cryoablation can trigger the release of damage-associated molecular patterns (DAMPs), including high mobility group box 1 (HMGB1) and heat shock proteins (HSPs), from injured cells (32, 33). These DAMPs act as danger signals that promote the recruitment and activation of antigen-presenting cells, thereby facilitating antigen-specific T-cell responses (34, 35). Additionally, cryoablation may modulate local immunosuppression. Preclinical studies suggest that cryoablation may reduce the abundance or suppressive activity of immunoregulatory cells such as regulatory T cells and myeloid-derived suppressor cells. Concurrently, cryoablation may increase the infiltration of effector cells, such as CD8^+^ T cells. These changes may contribute to a more permissive immune microenvironment for subsequent systemic antitumor responses (36). However, the extent and pattern of cryoablation may critically influence the magnitude and durability of the antitumor immune response. While complete ablation can eradicate the target lesion, it may also shorten local antigen exposure; in contrast, the sublethal peripheral zone of the cryolesion may be more immunogenic and may contribute more substantially to sustained adaptive immune priming and immune memory. Moreover, cryoablation is not uniformly immune activating, as both immunostimulatory and immunosuppressive effects have been reported depending on tumor type, freezing volume, residual tissue burden, and the local balance between pro-inflammatory and immunoregulatory signals (28, 32) (**Figure 1**).

### 2.3 Clinical application of cryoablation in NSCLC

As a minimally invasive technique, cryoablation is generally considered feasible and reasonably safe when performed in appropriately selected patients. The most common complications are pneumothorax and mild, self-limiting hemoptysis (37). Others, such as pleural effusion, puncture site pain, and transient post-procedural fever, are generally mild to moderate and manageable with standard perioperative care. The incidence of severe complications is low when performed by experienced operators following strict preoperative assessment (38).

In selected clinical settings, cryoablation may offer local tumor control for patients who are not candidates for surgery, although efficacy varies according to stage, lesion characteristics, and treatment intent (39). In advanced disease, it effectively reduces tumor burden in primary or oligometastatic lesions, rapidly alleviating symptoms like chest pain, cough, and hemoptysis, thereby playing a key role in palliative care and improving quality of life (40). In the immunotherapy era, interest in cryoablation has expanded beyond local tumor control because of its potential immunomodulatory effects. However, its precise role in multidisciplinary NSCLC management remains to be better defined (41, 42).

## 3 Immunotherapy in NSCLC: opportunities and limitations

Immunotherapy has become a central component of advanced non-small cell lung cancer (NSCLC) treatment and has substantially improved survival in selected patient populations. Immune checkpoint inhibitors (ICIs), particularly agents targeting programmed cell death protein 1 (PD-1), programmed death-ligand 1 (PD-L1), and cytotoxic T-lymphocyte-associated antigen 4 (CTLA-4), have reshaped the therapeutic landscape of advanced NSCLC. However, durable clinical benefit remains limited to a subset of patients. Primary and acquired resistance, heterogeneous biomarker performance, immune-related adverse events, and limited evidence in special patient populations continue to restrict the broader efficacy of immunotherapy (43). These limitations have stimulated growing interest in combination strategies capable of enhancing tumor immunogenicity and remodeling the tumor immune microenvironment. In this context, cryoablation has emerged as a potentially complementary local therapy because it may promote tumor antigen release, induce immunogenic cell death, and facilitate antitumor immune activation.

### 3.1 Rationale for immunotherapy in NSCLC

The rationale for immunotherapy is grounded in the dynamic interaction between tumor cells and host immunity, including immune surveillance, equilibrium, and immune escape (44, 45). Although NSCLC was once considered to have low immunogenicity, recent studies have revealed extensive immune cell infiltration in its tumor microenvironment, along with various immunosuppressive mechanisms used by tumor cells to evade immune detection. The primary goal of immunotherapy is to overcome this immune tolerance and restore effective antitumor responses.

In clinical practice, the efficacy of immunotherapy in NSCLC is limited by several factors. Only a subset of patients achieves clinical benefit due to low response rates and a lack of reliable biomarkers. Primary and acquired resistance are common, with some patients showing no initial response or progressing after an initial response. Additionally, immune-related adverse events can affect multiple organs and, in severe cases, be life-threatening. Since the introduction of immune checkpoint inhibitors, immunotherapy has moved from later-line use to a frontline option in selected NSCLC populations, reshaping the therapeutic landscape (46, 47). Nevertheless, its limitations remain a critical challenge in clinical practice and translational research.

### 3.2 Immune checkpoint blockade in NSCLC: clinical landscape, limitations, and rationale for cryo-immunotherapy

Immune checkpoint blockade has become a cornerstone of systemic therapy for advanced NSCLC without actionable driver alterations. Rather than broadly activating immunity, PD-1/PD-L1 and CTLA-4 inhibitors restore antitumor T-cell activity by interrupting inhibitory signals that contribute to T-cell exhaustion and impaired priming (48). In clinical practice, the relevance of these pathways is reflected less by their basic biology than by their role in defining modern first-line strategies, including ICI monotherapy for selected PD-L1-high tumors, ICI–chemotherapy combinations across histologic subtypes, and dual-checkpoint regimens in selected patients. However, only a subset of patients derives durable benefit, indicating that checkpoint blockade alone is often insufficient to convert a poorly inflamed or immunosuppressive tumor microenvironment into an effective antitumor immune response.

#### 3.2.1 Current clinical landscape of immune checkpoint inhibitors in NSCLC

Multiple phase 3 trials have established immune checkpoint inhibitors (ICIs) as standard-of-care in advanced NSCLC. In the first-line setting, pembrolizumab monotherapy improved survival in PD-L1-high tumors, while pembrolizumab plus chemotherapy extended benefit regardless of PD-L1 expression (46, 49–51). Combination regimens targeting both PD-1/PD-L1 and CTLA-4 have also shown efficacy: CheckMate-227 demonstrated superior overall survival with nivolumab plus ipilimumab in high-tumor-mutational-burden tumors, and CheckMate-9LA added two cycles of chemotherapy to this backbone (52, 53). In the anti-angiogenesis/ICI space, IMpower150 (atezolizumab plus bevacizumab and chemotherapy) showed improved progression-free and overall survival, whereas IMpower130 and IMpower110 further defined the role of atezolizumab in first-line and monotherapy settings, respectively (54–56). The POSEIDON trial evaluated tremelimumab plus durvalumab and chemotherapy, offering additional regimen options (57). Collectively, these trials have reshaped first-line treatment but also underscored persistent challenges: response rates remain modest (typically 30–50% even in biomarker-selected populations), primary resistance is common, and reliable predictive biomarkers beyond PD-L1 and tumor mutational burden are lacking.

#### 3.2.2 Key limitations and resistance mechanisms

Although ICIs have changed the treatment landscape of NSCLC, major limitations remain, including primary and acquired resistance, biomarker heterogeneity, and treatment-related toxicity. From a Chinese-population perspective, PD-L1 expression, the most important predictive biomarker, shows high inter- and intratumoral heterogeneity, and PD-L1 testing in China is still at an early stage with many practical challenges (58). Immune-related adverse events remain clinically relevant and may occasionally require treatment interruption, particularly in vulnerable populations (59). The role of ICIs in EGFR-mutant NSCLC remains poorly understood—a particular concern given the higher EGFR mutation rate in Chinese patients compared with Western populations (60). Furthermore, clinical data are lacking for special populations such as elderly patients, those with poor performance status, brain metastases, or autoimmune diseases, as these groups are often excluded from trials. These limitations underscore the need for novel combination strategies that can improve efficacy, overcome resistance, manage toxicity, and expand the benefit to understudied populations—a niche where cryoablation, by potentially modulating the tumor immune microenvironment, may play a role.

#### 3.2.3 Rationale for combining cryoablation with immune checkpoint blockade

Cryoablation may enhance the efficacy of immune checkpoint blockade by acting upstream in the cancer-immunity cycle. During freeze–thaw injury, tumor cells undergo necrosis and immunogenic cell death, leading to the release of tumor-associated antigens, neoantigens, and damage-associated molecular patterns. These signals may promote dendritic-cell recruitment, antigen uptake, and cross-presentation, thereby facilitating the priming and expansion of tumor-specific cytotoxic T cells (32). In this context, cryoablation may function as an *in situ* vaccination strategy, converting locally destroyed tumor tissue into a source of endogenous antigens (11). However, the immunologic outcome of cryoablation is not uniformly stimulatory; factors such as the volume of ablated tissue, freeze–thaw parameters, and the ratio of necrosis to apoptosis may tilt the balance toward immune suppression or tolerance. This provides a biological rationale for combining cryoablation with PD-1/PD-L1 blockade: cryoablation may increase antigen availability and immune priming, whereas checkpoint inhibition may sustain effector T-cell function and prevent tumor-mediated T-cell exhaustion. Although early clinical and translational studies have suggested immune activation after cryoablation-based combinations, including increased inflammatory cytokines and CD8^+^ T-cell infiltration, current evidence remains preliminary and requires validation in prospective trials.

### 3.3 Cellular immunotherapy: challenges and opportunities in combination with cryoablation

Beyond checkpoint blockade, cryoablation may also provide an immune-priming platform for cellular immunotherapy by increasing antigen release, inflammatory chemokine production, and effector-cell recruitment within the tumor microenvironment.

Cellular immunotherapy for NSCLC is primarily based on adoptive cell transfer technologies, including chimeric antigen receptor T-cell (CAR-T) therapy and natural killer (NK) cell therapy. CAR-T cells targeting tumor-associated antigens such as mesothelin, EGFR, and MUC1 have shown preclinical efficacy in lung adenocarcinoma models. Emerging strategies such as CAR-NK and CAR-M are being developed to overcome the immunosuppressive tumor microenvironment in solid tumors (61, 62). NK cell therapy offers the advantage of recognizing and killing tumor cells without the need for antigen priming. Preliminary clinical studies have demonstrated synergistic effects when NK cell therapy is combined with cryoablation (63). Cryoablation induces immunogenic cell death and releases large amounts of tumor antigens and damage-associated molecular patterns, which in turn enhance the homing ability and cytotoxic activity of infused immune cells (63, 64). Despite ongoing challenges—including an immunosuppressive tumor microenvironment, the need for CAR structure optimization, and limited efficacy as monotherapy—the combination of cellular immunotherapy with cryoablation represents a promising strategy to improve antitumor responses in NSCLC.

## 4 Mechanistic basis of cryo-immunotherapy synergy

Recent studies suggest that cryoablation may modulate the tumor immune microenvironment and thereby enhance the activity of systemic immunotherapy. For instance, cryoablation has been shown to reshape the immune landscape not only at the treated site but also in distal tumors, an effect associated with enhanced systemic antitumor immunity (65). This provides a biologically plausible rationale for combination strategies in advanced tumors. Mechanistically, cryoablation may initiate local immune activation, while immunotherapy may amplify and sustain the resulting systemic antitumor response. Building upon the cellular immunotherapy discussion above, the mechanistic synergy between cryoablation and immunotherapy warrants detailed exploration.

### 4.1 Immunological mechanisms underlying synergy

Recent mechanistic evidence suggests that cryoablation may induce features consistent with immunogenic cell death program characterized by DAMP release, partial preservation of tumor ultrastructure, dendritic-cell recruitment, and remodeling of both local and distal tumor immune microenvironments. The synergistic effect between cryoablation and immunotherapy is supported by a distinct biological foundation centered on cryoablation’s unique capacity to induce immunogenic cell death (ICD). Compared with some heat-based ablation modalities, cryoablation may better preserve native antigenic structures and induce an ICD-associated pattern of DAMP release (66).

First, cryoablation induces a cryo-specific ICD program. Key molecular hallmarks include surface exposure of calreticulin, release of HMGB1 and ATP, and passive release of heat shock proteins (e.g., HSP70) from disrupted cellular compartments (67). Importantly, cryoablation partially preserves tumor ultrastructure, enabling sustained antigen release that facilitates progressive uptake by antigen-presenting cells (APCs) (65). Second, cryo-induced DAMPs orchestrate APC recruitment and activation. The resulting inflammatory milieu—characterized by chemokines such as CCL2, CCL3, and CXCL10—promotes dendritic cell (DC) maturation and migration to draining lymph nodes, where they cross-present tumor antigens to naïve T cells, establishing an *in situ* vaccination effect. Third, this cryo-primed T cell response is often constrained by the immunosuppressive tumor microenvironment (TME). Immune checkpoint inhibitors (e.g., anti-PD-1/PD-L1) can overcome this limitation by releasing T cells from negative regulation (16, 67). Notably, cryoablation itself may transiently upregulate PD-L1 on residual tumor cells, providing a biological rationale supporting exploration of combination therapy.

One potentially important consequence of this synergy is the abscopal effect, although this phenomenon remains inconsistently observed in clinical settings. Mechanistically, tumor-specific T cells activated by cryoablation and sustained by checkpoint blockade may enter the systemic circulation and recognize metastatic lesions expressing shared antigens (68, 69). However, current evidence for the abscopal effect following cryoablation is largely derived from isolated case reports and preclinical models; its true incidence remains unknown due to the lack of standardized definitions and systematic reporting in clinical trials (70).

### 4.2 Evidence from preclinical models

Studies included in **Tables 1** and **2** were identified through searches of PubMed, Embase, Web of Science, and the Cochrane Library up to March 2026 using terms related to NSCLC, cryoablation, cryotherapy, immune checkpoint blockade, immunotherapy, and cryo-immunotherapy. Original preclinical and clinical studies directly relevant to cryoablation-based immunotherapy were prioritized, while reviews, editorials, duplicate publications, and studies lacking extractable mechanistic, efficacy, or safety outcomes were excluded.

**Table 1:** Preclinical evidence supporting cryo-immunotherapy in NSCLC-relevant models.

Study	Tumor model	Cryoablation Parameters	Co-administered treatment(s)	Immunotherapy regimen	Key immune findings	Main limitation
Li et al. (71)	LLC in C57BL/6N mice	Complete ablation; −40°C; 2 freeze–thaw cycles (60 s/60 s) via argon–helium cryotechnology	Cryoablation + PD-1 inhibitor	PD-1 inhibitor (HY-132192) 10 mg/kg by intraperitoneal injection on days 1, 4, 7, and 10 after cryoablation	Inhibited distant tumor growth, prolonged survival, reduced MDSCs, and increased CD8+ T-cell infiltration	Short follow-up; long-term tumor control unclear; All included studies use the LLC murine model
Liu et al. (72)	Bilateral LLC in C57BL/6N mice	Complete ablation; −140°C; 2 freeze–thaw cycles (60 s/60 s) via argon–helium cryotechnology	Cryoablation + PD-1 inhibitor	PD-1 inhibitor (HY-132192) 10 mg/kg (200 μg per mouse) by intraperitoneal injection on days 1, 4, 7, and 10 after cryoablation	Enhanced systemic antitumor immunity and prolonged survival; PI3K/AKT/mTOR pathway implicated	Murine model only; clinical translation uncertain; All included studies use the LLC murine model
Zhai et al. (73)	3LL in C57BL/6 mice	Tumor-centered cryoprobe; −75°C; single 30-s freeze via liquid nitrogen cryoablation	Cryoablation + Anti-PD-L1 antibody	Anti-PD-L1 monoclonal antibody (clone 10F.9G2) 150 μg per mouse by intraperitoneal injection twice per week, starting on day 8 after tumor inoculation	Restored PD-1+ CD8+ T-cell function; increased IFN-γ production and mitochondrial activity	Small sample size; limited statistical robustness; All included studies use the LLC murine model
Alteber et al. (74)	D122-luc-5.5 LLC in C57BL/6 mice	Liquid nitrogen-frozen probe; two 10-s freezes at 7-day interval; 5-mm ice ball	Cryoablation + Intratumoral DCs + CpG-ODN	Intratumoral injection of immature dendritic cells, 1 × 10^6^ cells per mouse, administered 1 hour after cryoablation, repeated at weekly intervals for 2 cycles	Strong inhibition of primary and metastatic tumors; robust Th1-biased CTL response and immune memory	CpG-ODN translational relevance to humans may be limited; All included studies use the LLC murine model
Machlenkin et al. (75)	D122 LLC in C57BL/6 mice	Liquid nitrogen-frozen tweezers; single 10-s freeze; 5-mm ice ball	Cryoablation + Intratumoral injection of 1×10^6^ immature dendritic cells	Peritumoral injection of immature dendritic cells, 4 × 10^6^ cells per mouse, immediately after cryoablation; followed by CpG-ODN 50 μg per mouse at 0, 6, 12, or 24 hours after dendritic cell injection	Robust CTL response, strongest inhibition of lung metastasis, generated effector memory CD8+ T cells	Ex vivo DC strategy may be difficult to standardize clinically; All included studies use the LLC murine model
Zhang et al. (76)	LLC in C57BL/6 mice	Argon-helium cryoablation; −140°C; 2 freeze–thaw cycles (20 s/10 s); 1.5–3 mm ice ball	Cryoablation + Peritumoral DCs + CpG-ODN (12h post-cryo)	Intratumoral co-administration of immature dendritic cells, 2 × 10^6^ cells per mouse, and CpG-ODN 10–50 μg per mouse, administered 1 hour after cryoablation, repeated at weekly intervals for 2 cycles	Optimal survival, strongest rechallenge resistance, highest Th1 cytokines, enhanced T cell proportions	Optimal timing identified in mice may not directly translate to humans; All included studies use the LLC murine model

LLC, Lewis Lung Carcinoma; PD-1, Programmed Cell Death Protein 1; MDSCs, Myeloid-Derived Suppressor Cells; PI3K, Phosphoinositide 3-Kinase; AKT, Protein Kinase B; mTOR, Mammalian Target of Rapamycin; NSCLC, Non-Small Cell Lung Cancer; 3LL, Lewis Lung Carcinoma (alternative designation); IFN-γ, Interferon-Gamma; CTL, Cytotoxic T Lymphocyte; DCs, Dendritic Cells; CpG-ODN, Cytidyl Guanosyl Oligodeoxynucleotide; TLR9, Toll-Like Receptor 9; Th1, T Helper Type 1. All reported temperatures refer to the set probe-tip temperature measured by the integrated thermocouple inside the cryoprobe.

**Table 2:** Clinical evidence of cryoablation-based immunotherapy strategies in NSCLC.

Study	Population	Clinical study design	PD-L1 status	EGFR/ALK status	Cryoablation-based strategy/comparator	ICI regimen	Main efficacy findings	Safety findings	Key limitations
Wang et al. (77)	90 patients with advanced NSCLC (Stage IIIB/IV)	Retrospective study	Group A (Cryo+Pembro, n=36): ≥50%: 9 (25.0%); 1-49%: 11 (30.6%); <1%: 4 (11.1%); Unknown: 12 (33.3%) Group B (Cryo alone, n=54): ≥50%: 20 (37.0%); 1-49%: 16 (29.6%); <1%: 8 (14.8%); Unknown: 10 (18.5%)	Not reported	Cryoablation + Pembrolizumab vs Cryoablation alone	Pembrolizumab 100 mg every 3 weeks; first dose within 2 weeks after cryoablation; continued until disease progression or intolerable toxicity	ORR: 75.0% vs 61.1% (p=0.045) mOS: 28.1 vs 24.2 months (p=0.009) mPFS: 12.8 vs 8.4 months (p=0.011)	No treatment-related mortality; toxicity manageable	Incomplete PD-L1 data; benefit interpretation may be confounded
Chen et al. (78)	108 patients with advanced NSCLC (stage IIIB-IV)	Retrospective study	Not reported	Not reported	Cryoablation + PD-1 inhibitor vs PD-1 inhibitor monotherapy	Sintilimab 200 mg every 3 weeks for 1–2 cycles as induction → cryoablation → Camrelizumab 200 mg every 3 weeks as maintenance, resumed within 7 days after cryoablation; continued until disease progression or up to 24 months	ORR: 75.0% vs 53.85% CR: 21.43% vs 3.85% PD: 0% vs 17.31%	Comparable AE rates; no unexpected toxicity	Incomplete AE grading; toxicity severity unclear
Zheng et al. (80)	60 patients with advanced NSCLC (Stage IIIB-IV)	Randomized Controlled Trial (Single-Center)	Not reported	Not reported	Cryoablation + Camrelizumab vs Camrelizumab + Chemotherapy	Camrelizumab 200 mg every 3 weeks, initiated within 7 days after cryoablation; total of 4 cycles	mOS: NR vs 10.3 months (HR = 0.45, P = 0.003) mPFS: 9.6 vs 8.3 months (HR = 0.48, P = 0.005) ORR: 73.33% vs. 53.33% (P = 0.108, NS) DCR: 90.00% vs. 83.33% (P = 0.447, NS) Higher CD4^+^ T cells & CD4^+^/CD8^+^ ratio; comparable safety	Comparable toxicity; no grade 4–5 AEs	Single-center open-label design; performance bias possible
Yang et al. (81)	Cryoablation (n=13) among 62 advanced NSCLC patients	Phase II Randomized Controlled Trial	Overall cohort: ≥50%: 13 (21.0%); 1-50%: 15 (24.2%); Negative: 16 (25.8%); Unknown: 18 (29.0%)	No activating EGFR/ALK mutations (required for enrollment)	Primary comparison: Ablation (cryo+thermal) + PD-1/L1 vs PD-1/L1 alone Exploratory subgroup: Cryoablation vs Thermal ablation	Camrelizumab, Tislelizumab, or Pembrolizumab (per standard dosing, every 3 weeks); immunotherapy held during ablation and resumed after post-ablation inflammation resolved; continued until disease progression, intolerable toxicity, or consent withdrawal	Primary analysis (ablation overall vs control): mPFS 26.7 vs 11.7 months (HR = 0.213, p<0.001); OS trend (HR = 0.242, p=0.036) Exploratory subgroup: mPFS: Not reached vs 22.4 months (p=0.011); IFN-α elevation trend (p=0.078)	Well tolerated; low grade ≥3 toxicity	Limited biomarker analysis; mechanistic depth reduced
Vasiliauskas et al. (82)	55 previously untreated patients with metastatic NSCLC	Single-center, prospective, randomized study	Cryotherapy group (n=24): <1%: 11 (45.8%); 1-49%: 7 (29.2%); ≥50%: 6 (25.0%) Control group (n=30): <1%: 12 (40.0%); 1-49%: 9 (30.0%); ≥50%: 9 (30.0%)	EGFR/ALK wild-type (inclusion criterion)	Bronchoscopic cryotherapy + pembrolizumab (± chemotherapy) vs pembrolizumab (± chemotherapy) alone	Pembrolizumab (standard dose) every 3 weeks, initiated 7 (± 1) days after cryotherapy; maximum 4 cycles; given as monotherapy if PD-L1 ≥ 50%, or combined with platinum-based chemotherapy if PD-L1 < 50%	ORR: 28.6% vs 23.1% (p=0.461) DCR: 95.2% vs 73.1% (p=0.049) Safe; no significant increase in irAEs	No serious bleeding or pneumothorax; irAEs comparable	Limited subgroup analysis; biomarker-specific interpretation restricted
Nian et al. (83)	A 66-year-old female with stage III PSC and obstructive atelectasis	Case Report	TPS = 95%	EGFR/ALK/ROS1/RET/PIK3CA wild-type; KRAS exon 2 mutation	Transbronchial Cryoablation + Camrelizumab	Camrelizumab 200 mg every 21 days; first dose 5 days after cryoablation; total of 4 cycles (combined with nab-paclitaxel + carboplatin for 1 cycle, followed by nab-paclitaxel alone for 3 cycles)	Partial Response after 4 cycles; reversed atelectasis; enabled effective systemic therapy	Severe pneumonitis reported	Concurrent chemotherapy confounds attribution of benefit
Feng et al. (86)	64 patients with advanced NSCLC	Retrospective study	Not reported	EGFR/ALK mutation as exclusion criteria	Cryoablation +Nivolumab vs Cryoablation alone	Nivolumab 3 mg/kg every 2 weeks, initiated after cryoablation; continued until disease progression or intolerable toxicity	Higher T cells (CD4^+^, CD8^+^), IL-2, TNF-β; manageable AEs	No treatment-related deaths; toxicity manageable	No nivolumab-only arm; additive benefit unclear
Bonnet et al. (87)	23 NSCLC patients (from a total cohort of 78 patients)	Retrospective, single-center cohort study	Not reported	Not reported	Cryoablation +Pembrolizumab/Nivolumab	Pembrolizumab, Nivolumab, Ipilimumab, Atezolizumab, or Durvalumab (heterogeneous regimens, specific dosing not consistently reported); immune checkpoint blocker administered within 90 days before or 30 days after ablation	Improved PFS in NSCLC vs other tumors (p=0.019); safe; benefit in oligo-persistence subgroup	Feasible; low complication rate	Non-standardized ablation margins; local control variability possible
Leppelmann et al. (100)	8 of 12 NSCLC patients received cryoablation(from a total cohort of 65 patients)	Retrospective Safety Analysis, Mixed-modality safety study (ICI + locoregional therapy)	Not reported	Not reported	Cryoablation + ICI (Nivolumab/Pembrolizumab/Atezolizumab)	Pembrolizumab, Nivolumab, Atezolizumab, or Ipilimumab (heterogeneous regimens, specific dosing not reported); immune checkpoint inhibitor administered within 90 days before or 30 days after procedure	No new/unmanageable safety risks; well-tolerated	No unexpected toxicity reported	No formal control arm; incremental safety risk unclear
Lin et al. (63)	60 patients with advanced (Stage III/IV) NSCLC	Retrospective study	Not reported	Not reported	Percutaneous Cryoablation + allogeneic NK cells vs Cryoablation alone	Not reported	RR: 63.3% vs 43.3% DCR: 83.3% vs 70% Elevated levels of NK/T cells & Th1 cytokines; improved QOL; Grade 1 AEs only	Grade 1 AEs only; no hematologic toxicity	Non-standardized NK product; reproducibility limited
Yuanying (79)	161 patients with metastatic NSCLC	Retrospective study	Not reported	Not reported	Cryotherapy + Chemotherapy + DC-CIK Immunotherapy	Not reported	mOS: 27 (triple) vs 8.5 months (chemotherapy alone, P<0.001) Cryoablation + Chemotherapy (mOS: 18 months), Cryoablation + Immunotherapy (mOS: 17 months) > Chemotherapy alone	No severe complications; fever manageable	Non-randomized allocation; substantial selection bias possible

NSCLC, non-small cell lung cancer; ORR, objective response rate; mOS, median overall survival; mPFS, median progression-free survival; PD-1, programmed death-1; CR, complete response; PD, progressive disease; NR, not reached; HR, hazard ratio; DCR, disease control rate; irAE, immune-related adverse event; PSC, pulmonary sarcomatoid carcinoma; IL-2, interleukin-2; TNF-β, tumor necrosis factor beta; QOL, quality of life; NK, natural killer; RR, response rate; DC-CIK, dendritic cell-cytokine induced killer.

These mechanistic concepts are supported by a growing body of preclinical evidence. These studies suggest that cryoablation combined with selected immunomodulatory strategies can enhance local and systemic antitumor immune responses in murine lung cancer models. **Table 1** summarizes key studies, whose core findings can be summarized as the following interconnected points:

First, preclinical studies suggest that combination therapy can enhance both local and systemic antitumor effects compared with monotherapy. Compared with monotherapy, the combination regimen significantly suppressed primary and distant tumor growth and prolonged host survival. For instance, in the Lewis lung cancer model, cryoablation combined with PD-1 inhibitors similarly demonstrated synergistic tumor suppression and survival benefits (71, 72). Notably, while combined anti-PD-L1 therapy did not significantly increase total intratumoral CD8^+^ T cell numbers, it specifically reactivated the PD-1^+^ CD8^+^ T cell subset. The observed synergistic effect was attributed to the improvement of mitochondrial metabolism and the enhancement of interferon-γ production (73).Second, combination therapy may remodel the immunosuppressive tumor microenvironment by reducing suppressive myeloid or regulatory T-cell populations, enhancing antigen presentation, and promoting effector T-cell infiltration and activation. On one hand, it directly reduces the infiltration and suppressive function of myeloid-derived suppressor cells (MDSCs) via downregulation of the JAK2-STAT3-S100A8/A9 signaling axis (71), while also decreasing the proportion of regulatory T cells (Tregs) (74). On the other hand, beyond relieving immunosuppression, combination therapy actively promotes immune activation and memory formation. In particular, cryoablation combined with dendritic cell vaccines elicits robust tumor-specific CTL responses and Th1 immune polarization, and generates a distinctive population of long-lasting effector memory CD8+ T cells that provide sustained protection against tumor rechallenge (74, 75). Research has indicated that administering CpG-ODN 12 hours after cryoablation and dendritic cell injection yields optimal outcomes. This regimen induces elevated levels of Th1 cytokines (e.g., IL-12, IFN-γ) and generates robust long-term antitumor memory (76). Third, the therapy activates key signaling pathways. The combination of PD-1 inhibitors with other agents has been shown to enhance CD8^+^ T cell infiltration and function by activating the PI3K/AKT/mTOR pathway (72).

Overall, preclinical studies support the biological plausibility of combining cryoablation with immunotherapy, but the magnitude and direction of immune effects appear to depend on tumor model, ablation extent, immune partner, and treatment timing. These findings support further clinical investigation while also underscoring the need for standardized protocols and prospective immune monitoring. These mechanistic insights provide a critical theoretical foundation and research basis for translating cryoablation combined with immunotherapy into clinical practice.

## 5 Clinical evidence of cryo-immunotherapy in NSCLC

Cryoablation, a localized tumor eradication technique, has gained prominence in recent years when combined with systemic immunotherapy as a frontier approach in NSCLC treatment. This combined strategy aims to leverage the dual advantages of local ablation and systemic immune activation to overcome the efficacy limitations of monotherapy. This section systematically reviews the clinical trial landscape of combined therapy based on existing evidence, evaluating its efficacy and safety.

### 5.1 Overview of clinical studies

The current clinical literature on cryo-immunotherapy in NSCLC remains heterogeneous. Most available studies are retrospective and single-center, while randomized evidence is still limited. In addition, substantial differences in disease stage, treatment line, comparator regimens, cryoablation technique, and immune partner complicate direct cross-study comparisons.

Currently, the majority of clinical studies evaluating cryoablation in combination with immunotherapy in NSCLC are retrospective analyses. However, several prospective randomized controlled trials have also emerged, providing preliminary evidence for the feasibility of this combination approach.

A substantial body of research has been dedicated to the analysis of cryoablation in conjunction with immune checkpoint inhibitors (ICIs, predominantly PD-1/PD-L1 inhibitors) for patients diagnosed with advanced NSCLC. For instance, the study by Wang et al., which included 90 patients with stage IIIB/IV disease, compared cryoablation combined with pembrolizumab to cryoablation alone (77). In contrast, the study by Chen et al., which included 108 patients, compared the combination therapy to immunotherapy alone (78). Preliminary studies have demonstrated the clinical efficacy of the combination regimen (77, 78). Additionally, the research scope has been expanded to encompass combinations with other immunotherapies. For instance, Lin et al. explored the use of cryoablation in combination with allogeneic NK cell infusion, while Yuanying et al. investigated a triple-combination regimen involving cryoablation, chemotherapy, and dendritic cell-cytokine-induced killer (DC-CIK) immunotherapy (63, 79).

It is important to note that higher-level evidence is being accumulated. Zheng et al. conducted a single-center randomized controlled trial comparing cryoablation plus PD-1 inhibitors versus PD-1 inhibitors plus chemotherapy in patients with advanced NSCLC (80). Separately, Yang et al. conducted a randomized phase 2 trial (BOOSTER) comparing ablation (cryoablation or thermal ablation) plus continued immunotherapy versus immunotherapy alone in patients with oligo-residual disease after anti-PD-1/L1 therapy (81). Vasiliauskas et al. conducted a prospective randomized study evaluating the efficacy of bronchoscopic cryotherapy combined with pembrolizumab-based systemic therapy (82). These studies provide higher-level evidence than retrospective analyses, although their sample sizes and single-center design still limit generalizability. Furthermore, case reports provide exploratory clinical observations in selected complex scenarios. For instance, Nian et al. reported a case of stage III pulmonary sarcomatoid carcinoma with atelectasis that was refractory to chemotherapy, where bronchoscopic cryoablation relieved airway obstruction. Subsequent combination therapy with a PD-1 inhibitor ultimately achieved partial remission. This case highlights the potential value of cryoablation in modifying the local tumor microenvironment and facilitating subsequent systemic therapy (83).

However, not all studies have shown favorable outcomes. In the CRYOVATE phase 1 trial, percutaneous cryoactivation followed by pembrolizumab yielded an ORR of only 25%, leading to early termination for futility (84). Similarly, a retrospective study of advanced soft tissue sarcomas found that while cryoablation achieved a 59.3% ORR in treated lesions, only 7.4% of untreated distant lesions regressed, arguing against a synergistic effect (85). These negative findings suggest that the immunological synergy between cryoablation and immunotherapy is not universal and may depend on tumor type, ablation parameters, treatment sequencing, and the balance between pro-inflammatory and immunosuppressive responses.

### 5.2 Clinical efficacy outcomes

Available clinical studies suggest that cryoablation combined with immunotherapy may improve treatment outcomes in selected patients with advanced NSCLC, although the current evidence remains heterogeneous.

A multitude of studies have reported that the combination therapy group exhibited superior outcomes in terms of objective response rate (ORR) and disease control rate (DCR) when compared to the control group. Wang et al. demonstrated a significantly higher ORR in the combination group (75.0% vs. 61.1%) (77). Lin et al. reported a DCR of 83.3% following NK cell combination therapy (63). Despite the fact that the prospective study by Vasiliauskas did not demonstrate a statistically significant improvement in ORR, the combination group exhibited a significantly higher DCR of 95.2% in comparison to the control group, which registered at 75% (82).

Several studies have reported potential improvements in progression-free and overall survival; however, these findings should be interpreted cautiously given the heterogeneity of study design and comparator regimens. Retrospective analyses by Wang et al. demonstrated significant median PFS (12.8 vs. 8.4 months) and OS (28.1 vs. 24.2 months) advantages in the combination group (77), whereas Chen et al. reported improved response rates without formal survival analysis (78). In a randomized controlled trial, Zheng et al. reported significant improvements in median PFS (9.6 vs. 8.3 months) and OS (not reached vs. 10.3 months) for the cryoablation plus PD-1 inhibitor group versus the control arm, which consisted of camrelizumab plus chemotherapy. However, this comparison should be interpreted as cryoablation plus camrelizumab versus a chemotherapy-containing camrelizumab regimen, rather than as evidence that cryoablation improves outcomes over ICI monotherapy (80). In the BOOSTER trial, mixed local ablation, including cryoablation and thermal ablation, combined with continued PD-1/PD-L1 inhibitor therapy prolonged median PFS compared with continued immunotherapy alone. Because the primary efficacy analysis pooled different ablation modalities and only a subset of patients received cryoablation, these results should not be interpreted as cryoablation-specific evidence. The exploratory cryoablation subgroup finding should be considered hypothesis-generating because of the small sample size and baseline imbalance (81). Yuanying’s retrospective analysis further demonstrated that combination regimens incorporating cryoablation (whether with chemotherapy or immunotherapy) yielded significantly superior median OS compared to their cryoablation-free counterparts (79).

With respect to the exploration of mechanisms, certain studies have noted positive alterations in peripheral immune markers subsequent to combination therapy, including increased CD4^+^ T cells, CD8^+^ T cells, NK cell counts, and Th1-type cytokine levels (e.g., IL-2, IFN-γ). Collectively, these observations provide preliminary clinical support for the hypothesis that cryoablation-based combination strategies may enhance systemic antitumor immunity.

### 5.3 Safety and adverse events

The safety profile of combination therapy warrants close attention, primarily concerning the potential additive risks of cryoablation-related complications and immune checkpoint inhibitor-related adverse events (irAEs).

Available data suggest that the combination of cryoablation and immunotherapy is feasible and has not revealed major unexpected safety signals, although current safety conclusions remain limited by study size and design. Cryoablation-related complications are generally mild to moderate and frequently transient. Common complications include puncture site pain, bleeding, hematoma, pneumothorax, and others. As shown in **Table 2**, multiple studies have reported no treatment-related fatalities or unmanageable severe complications associated with cryoablation (e.g., Yuanying, Yang, Vasiliauskas). Furthermore, bronchoscopic cryotherapy exhibited a favorable safety profile.

Regarding the safety profile, although the robust inflammatory response induced by cryoablation may theoretically increase the risk of irAEs, current clinical evidence has not demonstrated a significant difference in the incidence of irAEs compared to other ablation modalities. For instance, Feng et al. found no significant difference in adverse event rates between the combination group and the control group (86). Vasiliauskas’ prospective study specifically evaluated this risk, finding that the incidence of irAEs (25.0%) after combined cryotherapy was comparable to that in the systemic therapy-only group (26.7%) (82). Bonnet et al. also reported that the combination regimen was both feasible and safe, with no unexpected toxicities (87).

Overall, existing studies support the feasibility of combining cryoablation with immunotherapy, without evidence of unexpected safety signals in current studies. However, current safety evidence remains limited by small sample sizes, retrospective designs, and inconsistent adverse-event reporting, underscoring the need for larger prospective studies with longer follow-up.

### 5.4 Challenges in optimizing and standardizing cryo-immunotherapy

One of the major obstacles to optimizing cryo-immunotherapy in NSCLC is the lack of standardized cryoablation parameters, as variables such as freeze–thaw cycles, minimum temperature, ice ball coverage, freeze duration, and the timing of immunotherapy all influence the antitumor immune response. Although double-cycle protocols persist in some settings, triple freeze–thaw cycles have emerged as the standard of care in high-volume institutions (27). The ice ball periphery (−20 °C to 0 °C) has been proposed as a key immunologically active zone, where cells undergo immunogenic cell death and serve as a sustained source of tumor antigens; this has prompted the hypothesis that partial ablation may be preferable to complete ablation when combined with ICIs, though this has yet to be validated in prospective trials (87, 88). Rapid freeze–thaw kinetics are thought to enhance membrane disruption, but the optimal duration must balance immunogenicity against the risk of injury to adjacent normal structures. Similarly, the optimal timing of ICI administration relative to cryoablation remains unclear, though preclinical data suggest that a window of several days post-ablation may coincide with peak dendritic cell maturation and T-cell priming (89). Finally, the operator- and device-dependent nature of the procedure introduces additional variability that complicates multicenter efforts and limits generalizability. Addressing these gaps through well-designed, prospective studies with standardized, harmonized protocols will be essential to define evidence-based practice and enable meaningful cross-trial comparisons.

## 6 Discussion

Cryoablation combined with immunotherapy represents a promising emerging strategy in the treatment of non-small cell lung cancer (NSCLC). While this synergistic approach holds significant promise, its transition into a standardized clinical protocol necessitates addressing several critical scientific and technical challenges (23, 90).

The rationale for this combination lies in the potential of cryoablation to initiate local immune activation that may be amplified by systemic immunotherapy (91). By inducing immunogenic cell death (ICD) while preserving the native conformation of tumor antigens, cryoablation functions as an *in situ* vaccine. This process initiates the cancer–immunity cycle, which is subsequently amplified and sustained by immune checkpoint inhibitors (ICIs) that relieve the functional suppression of effector T cells (67). Mechanistically, cryoablation may initiate antigen release, DAMP-mediated dendritic-cell activation, and local immune remodeling, while checkpoint blockade may sustain effector T-cell function and systemic antitumor immunity. The timing and biological context of this combination may influence whether local immune priming translates into systemic antitumor responses and durable immune memory (92).

Despite this promising mechanistic basis, several key obstacles hinder clinical translation. A pivotal challenge remains the determination of the optimal therapeutic sequence. The timing of ICI administration relative to cryoablation may profoundly influence the kinetics of antigen release, dendritic cell maturation, and T-cell priming (93). Preclinical evidence indicates that the sequence of administration is critical for maximizing efficacy; however, the absence of large-scale prospective studies has left a gap in unified clinical standards. For instance, performing cryoablation prior to immunotherapy may facilitate pre-release of antigens, thereby establishing an immunological foundation for subsequent immune checkpoint blockade. Conversely, concurrent administration may leverage ICIs to prevent T-cell exhaustion induced by massive antigen exposure following ablation. These sequencing considerations are mechanistically underscored by the observation that the intact tumor and its draining lymph nodes serve as a critical reservoir of tumor-specific tissue-resident memory T (T_RM) cells; surgical removal of this reservoir prior to immunotherapy may deplete a substantial proportion of antitumor immunity, thereby compromising ICI efficacy (94). Future research must prioritize identifying the optimal window for intervention—whether as a neoadjuvant priming phase or a concurrent strategy to prevent post-ablation T-cell exhaustion—and integrate pharmacokinetic and immunodynamic monitoring data to enable personalized sequencing decisions.

The identification of reliable biomarkers will be important for patient selection and response monitoring in cryo-immunotherapy. Traditional markers, such as PD-L1 expression, often fail to accurately predict responses to this multimodal regimen. Dynamic biomarkers derived from liquid biopsy demonstrate significant value. In a study of stage I NSCLC patients treated with microwave ablation, ctDNA positivity rates increased from 41.9% at baseline to 60.7% within one hour post-ablation, then declined to 34.6% at follow-up. Notably, patients who achieved ctDNA clearance had outcomes comparable to those with persistently undetectable ctDNA (95). Collectively, these data suggest that ctDNA dynamics may serve as a potential real-time surrogate for molecular response and minimal residual disease. Furthermore, peripheral blood T-cell receptor repertoire diversity reflects systemic immune reconstitution. Clonal expansion in this context may indicate an effective *in situ* vaccination effect (96). Additional indicators also hold potential value. These include circulating tumor cells, exosomes, cytokine profiles, and immune cell subsets. Integrating multi-omics data to construct composite biomarker models may facilitate the identification of patient subgroups. These subgroups are most likely to benefit from combination therapy (97).

Current evidence suggests that the overall safety profile of cryoablation combined with immunotherapy is encouraging, although it remains incompletely characterized. Nevertheless, the inflammatory activation induced by extensive cell lysis necessitates rigorous monitoring for severe immune-related adverse events. Mechanistically, the local inflammatory response elicited by cryoablation may amplify the systemic immune activation effects of immune checkpoint inhibitors. This theoretically increases the risk of serious adverse events. These events include immune-mediated pneumonitis and myocarditis (98, 99). Accordingly, baseline risk assessments should be conducted prior to treatment. Dynamic monitoring of inflammatory and immune markers should be performed during therapy (100). Standardized protocols for the grading and management of adverse events should also be established. Moreover, the lack of gold standards for ablation parameters contributes to treatment heterogeneity. These parameters include the number of probes, ice ball coverage, and number of freeze–thaw cycles. Standardizing these parameters is critical to ensuring a valid correlation between the intensity of local tissue destruction and the magnitude of the immune response. Efforts should thus be directed toward developing evidence-based operational guidelines for cryoablation techniques (92).

Future NSCLC management is likely to become increasingly multimodal and biomarker-informed. Current ClinicalTrials.gov entries suggest that cryoablation-based immunotherapy remains an early investigational strategy, with only a limited number of NSCLC studies identified, including NCT06580665, NCT07064876. Therapeutically, triple-combination strategies incorporating anti-angiogenic agents or novel immunomodulators may help overcome immune resistance, while advances in molecular imaging may enable non-invasive monitoring of the immune microenvironment (101, 102). Ultimately, rigorously designed multicenter randomized trials with standardized ablation protocols and prospective biobanking will be required to determine whether cryo-immunotherapy can become a clinically validated option for selected NSCLC populations.
